# The Role of Vitamin D in Modulating the Innate Immune Response in Children with Vesicoureteral Reflux

**DOI:** 10.3390/children13060811

**Published:** 2026-06-12

**Authors:** Marius-Cosmin Colceriu, Diana Jecan-Toader, Paul Luchian Aldea, Bogdan Bulată, Dan Delean, Alina Grama, Alexandra Mititelu, Tudor Lucian Pop, Simona Clichici, Teodora Mocan, Andreea-Liana Boț (Răchişan)

**Affiliations:** 1 Discipline of Physiology, Department of Functional Biosciences, “Iuliu Hațieganu” University of Medicine and Pharmacy, 400006 Cluj-Napoca, Romania; 2 Second Pediatric Clinic, Emergency Clinical Hospital for Children, 400177 Cluj-Napoca, Romania; 3 Medical Oncology Discipline, Department of Oncology, “Iuliu Hatieganu” University of Medicine and Pharmacy, 400012 Cluj-Napoca, Romania; 4 Discipline of Public Health and Management, Department of Community Medicine, “Iuliu Haţieganu” University of Medicine and Pharmacy, 400006 Cluj-Napoca, Romania; 5 Pediatric Nephrology, Dialysis and Toxicology Clinic, Emergency Clinical Hospital for Children, 400177 Cluj-Napoca, Romaniaandreea_rachisan@yahoo.com (A.-L.B.); 6 Second Pediatric Discipline, Department of Mother and Child, “Iuliu Haţieganu” University of Medicine and Pharmacy, 400177 Cluj-Napoca, Romania; 7 Nanomedicine Department, Regional Institute of Gastroenterology and Hepatology, 400158 Cluj-Napoca, Romania; 8 Faculty of Nursing and Health Sciences, Department 2, University of Medicine and Pharmacy “Iuliu Hatieganu” Cluj-Napoca, 400023 Cluj-Napoca, Romania

**Keywords:** vitamin D, vesicoureteral reflux, renal scars, IL-6, LL-37, NGAL, children

## Abstract

**Highlights:**

**What are the main findings?**

• In 25 children with vesicoureteral reflux, lower vitamin D levels tended to be seen in those with more recurrent acute pyelonephritis, and patients with renal scars had lower mean vitamin D than those without scars, although these differences were not statistically significant.
• Serum vitamin D showed a strong positive correlation with urinary LL-37/creatinine, but no meaningful association with urinary IL-6 or NGAL.
• The study suggests that vitamin D may be linked to urinary innate immune defense mainly through LL-37 modulation.

**What are the implications of the main findings?**

• Maintaining adequate vitamin D status may help reduce recurrent urinary infections in children with vesicoureteral reflux, especially in those with a deficiency.
• Vitamin D could be a potential adjunct strategy to support innate immunity and possibly lower the risk of renal scarring and reflux nephropathy, but larger prospective studies are needed to confirm this.

**Abstract:**

**Background**: Vitamin D, through its role in antimicrobial peptide (AMP) expression, may influence innate immunity and inflammation in urinary tract infections (UTIs). This study evaluated its role in patients with vesicoureteral reflux (VUR) and its contribution to the pathophysiology of reflux nephropathy (RN). **Methods**: We conducted a cross-sectional observational study of 25 pediatric patients with VUR, representing a subgroup analysis of a larger cohort examined in a previous study. We determined patients’ vitamin D status, correlated it with recurrent UTIs and RS, and explored its relationship with urinary LL-37, NGAL, and IL-6 levels as markers of innate immune function. **Results**: Serum vitamin D levels ranged from 10.7 to 123.2 ng/mL (mean 39.5 ng/mL); 12% had deficiency and 20% had insufficient levels. Low vitamin D levels were detected in patients with more than five acute pyelonephritis (APNs), with a mean value classified as insufficient (27.3 ng/mL). Patients with RS had a lower mean vitamin D level compared to those without (30.51 ng/mL vs. 41.23 ng/mL), though the difference was not statistically significant (*p* = 0.39). No significant associations were found between vitamin D and urinary IL-6 or NGAL levels. A strong positive correlation was observed between vitamin D and urinary LL-37/creatinine (r = 0.78, r^2^ = 0.61). **Conclusions**: Vitamin D appears to influence the frequency of UTIs and the development of RS, primarily by modulating LL-37 secretion, suggesting a possible role in the pathophysiology of RN.

## 1. Introduction

Vitamin D, also known as calciferol, is a fat-soluble secosteroid vitamin that is recognized as a pleiotropic modulator of biological functions [1]. It has long been recognized as a key regulator of calcium and phosphorus homeostasis. In recent years, studies have also highlighted its immunomodulatory role, including effects on cell differentiation and innate immune responses through vitamin D receptor expression in immune cells [2,3].

The influence of vitamin D on the innate immune system began to be understood when research revealed the presence of the vitamin D receptor in nearly all immune cells, including B lymphocytes, T lymphocytes, neutrophils, and antigen-presenting cells (dendritic cells and macrophages) [4]. Therefore, vitamin D’s antibacterial, antiviral, and antifungal effects are attributed to its immunomodulatory role, particularly through modulation of antimicrobial peptide (AMP) expression, with cathelicidins and defensins being among the most important. In vitro experiments have shown that vitamin D strongly induces the transcription of cathelicidins in epithelial and myeloid cells, while it acts as a secondary modulator of defensin expression, which is primarily stimulated by other immune modulators, such as IL-1β [5].

Cathelicidins are peptides that have been well-conserved throughout evolution, with the human species presenting a single type, namely LL-37. Upon contact with the bacterial membrane, LL-37 adopts an alpha-helix structure with amphipathic properties, its main activity being the lysis of the bacterial membrane through interaction with hydrophobic and phospholipid components [6]. At the urinary tract level, the urothelium and intercalated cells can synthesize LL-37 immediately after bacterial invasion, significantly increasing its concentration in urine during infections. In the more advanced stages of the infectious process, neutrophils become the primary site of LL-37 synthesis. As a key component of innate immunity, LL-37 helps protect the urinary tract by reducing microbial adherence to epithelial surfaces, interfering with biofilm development, enhancing phagocytic mechanisms, and attracting inflammatory cells, including monocytes and neutrophils. Furthermore, it displays direct antimicrobial activity against a wide range of Gram-positive and Gram-negative pathogens [6,7].

Given that one of the main sites of LL-37 synthesis in the kidney is represented by intercalated cells, certain pathologies associated with structural renal changes could influence LL-37 production and, consequently, susceptibility to infections. Clinical observations support the increased frequency of acute pyelonephritis (APNs) in patients with conditions that cause structural and functional changes in the collecting ducts, such as high-grade vesicoureteral reflux (VUR), obstructive uropathies, polycystic kidney disease, or renal dysplasia. To accurately determine LL-37 expression profiles in these pathologies and to identify new possibilities for augmenting its synthesis, further studies are needed [8,9].

Thus, given the importance of LL-37 in the initial stage of the innate immune response and its potential use as a prophylactic and therapeutic agent in urinary tract infections (UTIs), several studies conducted so far have sought to identify methods for exogenous stimulation of LL-37 synthesis. To date, vitamin D, short-chain fatty acids (butyric acid and phenylbutyrate), estrogens, and zinc have been identified as potential stimulators of AMP synthesis [8,10].

In the context of the increasing phenomenon of pathogen resistance to antibiotic therapies, identifying new agents with antibacterial activity and studying them for implementation in clinical practice is of great interest. This aspect is particularly important among patients with VUR, who require prolonged prophylactic antibiotic therapies and repeated curative antibiotic treatments in the context of recurrent UTIs, to prevent progression to reflux nephropathy (RN) and renal function impairment. For these reasons, the present study aims to comprehensively analyze AMP and vitamin D in patients with VUR.

One primary objective of the study was to determine vitamin D levels in patients with VUR and correlate these levels with the number of recurrent UTIs the patient has had up to the time of evaluation. The susceptibility of patients with vitamin D deficiency to recurrent UTIs may have implications for the development of renal scarring (RS) and, consequently, the pathophysiology of RN. For this reason, the study also assessed the correlation between vitamin D levels and the presence of RS and chronic kidney disease (CKD).

In light of the effect of vitamin D on the gene expression of AMP and to evaluate its modulatory function on the innate immune system, another objective of the study was to correlate vitamin D levels with urinary concentrations of LL-37, NGAL, and IL-6. Establishing these correlations will help us better understand the involvement of vitamin D in the innate immune response in the urinary tract and may guide future research directions.

## 2. Materials and Methods

We conducted a cross-sectional observational study of 25 pediatric patients with VUR, representing a predefined subgroup analysis derived from a previously published cohort examined in our earlier study [11].

The patients were recruited from the Pediatric Nephrology, Dialysis, and Toxicology Department of the Emergency Clinical Hospital for Children in Cluj-Napoca, Romania. The study duration was three years, with the patient selection and medical data collection period spanning from August 2021 to September 2024.

We enrolled in the study patients diagnosed with VUR through retrograde voiding cystourethrography, for whom the serum vitamin D level was determined at the same time as the urine sample collection for the determination of LL-37, NGAL, and IL-6. Only patients who had not experienced a UTI for at least one month before the urine sample collection were included. We excluded patients with spontaneously remitting VUR or VUR corrected surgically, as well as those for whom the serum vitamin D level was determined at a different time than the urine sample collection. Other exclusion criteria included a proven UTI following urine examination, the presence of an acute infectious or inflammatory process clinically or paraclinically proven, the absence of a clear history recorded in the medical documents regarding the number of UTIs in the past, and the presence of signs and symptoms of rickets or malnutrition. Thus, before inclusion in the study, each patient was clinically assessed, and to exclude any inflammatory or infectious process, we determined the following paraclinical parameters: leukocyte count with leukocyte formula, C-reactive protein, procalcitonin, erythrocyte sedimentation rate, and urine examination (dipstick, sediment, and culture). Initially, we identified 39 patients with active VUR, but after applying the inclusion/exclusion criteria, we established a final group of 25 patients. A comparison between the initial cohort (*n* = 39) and the final study group (*n* = 25) showed no statistically significant differences in key demographic (age, sex) or clinical parameters (VUR grade, number of APNs). Therefore, the final group can be considered representative of the original cohort.

The study was initiated after obtaining approvals from the ethics committees of the Cluj-Napoca Emergency Clinical Hospital for Children (82SC/20.05.2021) and the “Iuliu Hațieganu” University of Medicine and Pharmacy, Cluj-Napoca (168/28.05.2021). At the time of patient admission, we obtained informed consent from the parents or legal guardians, depending on the situation, to allow participation in the study and access to the medical data from the patient’s history. For children over the age of 7, we drafted a separate consent form to obtain their agreement to participate in the study. The informed consent forms for patients were drafted, along with an informational letter about the study, for two age groups (7–13 years and 14–18 years), ensuring they were suitable for their level of understanding.

This observational study was conducted and reported in accordance with the STROBE guidelines. As it represents a subgroup analysis of a previously published cohort and did not involve any interventional procedures, the study protocol was not subject to prior formal registration.

The patients’ medical history was obtained by consulting their medical records in the hospital’s archives and accessing the Hospital’s electronic application. We performed an analysis of the patients’ demographic characteristics (age, gender, nutritional status according to CDC criteria, and place of origin); clinical parameters; comorbidities; the number of UTIs in the past; the presence of RS, CKD, and albuminuria; as well as the history of therapeutic interventions (antibiotic prophylaxis, anti-reflux substance injection, and surgical interventions). Information regarding vitamin D supplementation, dietary intake, and sun exposure was not systematically available for all patients and, therefore, could not be consistently analyzed.

The diagnosis of VUR was established for each patient included in the study through retrograde voiding cystourethrography. This investigation was performed after the prior exclusion of UTIs. It was performed by bladder catheterization, followed by the introduction of an iodine-based contrast agent (Iohexol-Omnipaque) and repeated radiological exposures. The radiological examinations were performed using a Siemens Luminos radiology machine.

RS were detected through scintigraphic examination with DMSA (99mTc-dimercaptosuccinic acid). The diagnosis of RS was made based on the scintigraphic appearance of cortical defects in tracer uptake. Additionally, the scintigraphic appearance was correlated, where applicable, with the presence of cortical indentations detected during ultrasound evaluation. RN was defined as chronic renal damage associated with VUR, including patients with RS as well as those presenting other imaging or functional abnormalities suggestive of reflux-related kidney injury, such as reduced kidney size, abnormal scintigraphic findings, or impaired renal function. To ensure accurate evaluation of tracer uptake by the renal parenchyma, scintigraphic assessments were performed at least 3 months after an APN [12,13].

The diagnosis of APN was established based on the following clinical and paraclinical criteria: fever (above 38 °C), leukocyturia (positive test for leukocyte esterase on urine dipstick examination or detection of more than 10 leukocytes per field of view on microscopic examination of urinary sediment), positive nitrites on urine dipstick, positive urine culture (>100,000 CFU/mL), and presence of bacterial inflammatory syndrome (C-reactive protein > 2 mg/dL, procalcitonin > 1 ng/mL, and leukocytosis over 12,000/μL with neutrophilia). The number of APNs in the medical history of each patient was recorded in the database according to the data provided by the parents and those documented in the medical records based on tests conducted in our clinic. To facilitate the statistical analysis of the number of APNs, patients were categorized into four groups: 0 APNs, 1–2 APNs, 3–5 APNs, and >5 APNs.

In the category of CKD with impaired renal function, we included patients in stages 2–5 of CKD, meaning those with eGFR < 90 mL/min/1.73 m^2^. For patients under 16 years of age, we used the modified pediatric Schwartz formula (bedside) to calculate eGFR: k × height in cm/serum creatinine in mg/dL (k = 0.413 for children > 1 year, k = 0.45 for full-term infants ≤ 1 year, and k = 0.33 for preterm infants ≤ 1 year). For patients over 16 years of age, we used an online tool that employs the CKD-EPI 2021 equation based on serum creatinine. For patients under 2 years of age, due to the physiological characteristics of renal function, we interpreted eGFR values as normal, moderately reduced, or severely reduced, based on the reference values for age proposed by Schwartz and Furth [14]. These aspects regarding eGFR calculation and CKD stage determination are in accordance with KDIGO 2012/2021 guidelines [15,16].

Urine samples were collected in the morning, during the first micturition of the day. For older patients with complete bladder control (usually over the age of 2 years), urine samples were obtained using the midstream urine collection technique after local disinfection. In children under 2 years of age or those without adequate sphincter control, urine collection bags were used. These bags were applied by medical staff after thorough local disinfection. In exceptional cases where urine could not be collected using the methods mentioned above, bladder catheterization was performed using appropriately sized Foley catheters for the patient’s age. Within a maximum of 2 h after collection, the urine samples were evaluated through general urine analysis, microscopic examination of the sediment, and urine culture (with results read 48 h after culture plating). Additionally, urinary creatinine concentration was measured. Subsequently, the urine samples were centrifuged at 3500 revolutions per minute for 5 min and stored at −20 °C. Samples kept for more than a month were maintained at −80 °C. At the same time, venous blood samples were collected for a complete blood count, erythrocyte sedimentation rate, C-reactive protein, and serum creatinine determination. For measuring serum vitamin D levels, venous blood was collected in a vacutainer without anticoagulant, with a separator gel, and the serum was separated by centrifugation. Vitamin D determination was performed immediately after collection, without the need for freezing the samples for later analysis.

Hematological parameters were determined by flow cytometry using the Mindray LabXpert 6800 analyzer. Serum creatinine was determined by spectrophotometry, and C-reactive protein by turbidimetry using the Mindray SAL 6000 (BS800+CL2000) analyzer. The erythrocyte sedimentation rate was determined by capillary photometry using the Alifax SIRE Roller 20–LC analyzer. Procalcitonin was measured, where applicable, by chemiluminescence using the Mindray CL–1200i analyzer. The urine analysis was performed by reflectometry using the BioMaxima BM500 analyzer. Urinary sediment was analyzed by optical microscopy. Serum vitamin D levels were measured using standardized chemiluminescence immunoassay platforms (DiaSorin LIAISON XL and Mindray SAL 6000) routinely employed in our institutional laboratory, following the same internal quality-control and calibration protocols to ensure inter-assay comparability.

In accordance with the values provided by the Central Laboratory of Medical Analyses of the Cluj-Napoca Emergency Clinical Hospital for Children, Table 1 presents the reference intervals for serum vitamin D levels used in this study.

The evaluation of urinary biomarkers involved measuring the urinary concentrations of LL-37, IL-6, and NGAL. Before these determinations, the urine samples required gradual thawing by passive warming at room temperature (20–25 °C). To determine the urinary concentrations of LL-37, NGAL, and IL-6, we used human ELISA kits produced by Elabscience, Houston, TX, USA. Biomarker concentrations were determined using commercially available ELISA kits based on the sandwich immunoassay technique. The assays were performed on microplates pre-coated with antibodies specific for LL-37, IL-6, and NGAL. All procedures were conducted in accordance with the manufacturer’s protocols and accompanying instructions. Optical density measurements were obtained using an automated microplate reader (Dynex DS2, Dynex Technologies Inc., Chantilly, VA, USA). The analytical sensitivities of the assays were 0.129 ng/mL for LL-37, 0.095 pg/mL for IL-6, and 0.116 pg/mL for NGAL. To obtain comparable results, avoid dilution effects, and standardize the samples, we reported the measured values to the urinary creatinine, which was determined by spectrophotometry using the KONELAB 60i analyzer.

All data obtained were entered into a database using Microsoft^®^ Excel for Mac, Version 16.88 (24081116). For statistical analysis, we used IBM SPSS Statistics software, Version 29.0.2.0. For variables with continuous distributions, descriptive statistics were applied to calculate the means and standard deviations. The distribution of continuous variables was assessed using the Shapiro–Wilk test. Comparisons between two independent groups were performed with the Mann–Whitney U test. Differences in serum vitamin D concentrations across multiple groups were examined by one-way analysis of variance (ANOVA), followed by Tukey’s honestly significant difference (HSD) post hoc test when appropriate. Associations between continuous variables were investigated using Pearson’s correlation coefficient (r). Linear regression plots were generated to visualize these relationships, and the coefficient of determination (r^2^) was used to estimate the proportion of variance explained by the regression model. Post hoc power analyses were performed for the main correlation analyses to assess the statistical power of the observed associations. The dataset was additionally reviewed for potential influential outliers. In all statistical analyses performed, a *p*-value of less than 0.05 was considered statistically significant.

## 3. Results

In this study, 39 patients with VUR were included, from whom urine and blood samples were collected. After clinical and biological evaluation and use of the inclusion/exclusion criteria, the final study group consisted of 25 patients. Their ages ranged from 4 months to 16 years and 4 months. Regarding gender distribution, 13 patients were male, and 12 were female. As previously reported in the original cohort study, no significant differences in nutritional status were observed between patients and controls [11]. For most patients, the place of origin was urban, with an urban-to-rural ratio of 14-to-11. The same 14-to-11 ratio was recorded for the bilaterality of VUR, with 14 patients having bilateral involvement and 11 unilateral involvement. Regarding VUR complications, five patients (20%) showed RS on DMSA scintigraphy, 20 patients (80%) fulfilled the diagnostic criteria for RN, and one patient (4%) was diagnosed with high blood pressure. Following CKD staging, 22 patients (88%) were classified as stage 1, and three patients (12%) were classified as stage 2.

The serum level of 25-hydroxyvitamin D ranged from 10.71 ng/mL to 123.2 ng/mL, with an average value of 39.51 ng/mL. No patient presented severe vitamin D deficiency; three patients (12%) had vitamin D deficiency, five patients (20%) had insufficient levels, 16 patients (64%) had sufficient levels, and one patient (4%) had toxic levels.

Regarding the recurrence of UTIs in these patients, three patients (12%) had no UTI history, 6 patients (24%) had between one and two episodes of APNs, 7 patients (28%) had between three and five episodes of APNs, and 9 patients (36%) had more than five episodes of APNs.

The analysis of the correlation between serum vitamin D levels and the number of APNs revealed the lowest mean vitamin D values in the group of patients who had more than five episodes of APNs. This group’s mean vitamin D value falls within the insufficient level range (27.3 ng/mL). Patients with 3–5 episodes of APNs in their history had higher mean vitamin D levels than those with more than five APNs but lower than those with 1–2 APNs. The mean values for each group are presented in Table 2. The ANOVA test to analyze these differences between the four groups revealed that these differences are not statistically significant (*p* = 0.07).

Patients with RS had an average vitamin D level of 30.51 ng/mL, while those without RS had an average vitamin D level of 41.23 ng/mL (Table 3). However, this difference between the two groups was not statistically significant (*p* = 0.39).

Comparing patients with RNs and those without, we obtained mean vitamin D levels similar to the overall average for the entire study group. Thus, patients with RN had a mean vitamin D level of 39.46 ng/mL (SD 22.74), while those without had an average of 39.72 ng/mL (SD 22.69). There was no statistically significant difference between the two groups (*p* = 0.98).

In the analysis of serum vitamin D levels in relation to CKD stages, we recorded a mean vitamin D level of 39.92 ng/mL in patients with CKD stage 1 and a mean of 36.58 ng/mL in patients with CKD stage 2. This difference was not statistically significant (*p* = 0.67).

**Figure 1:**
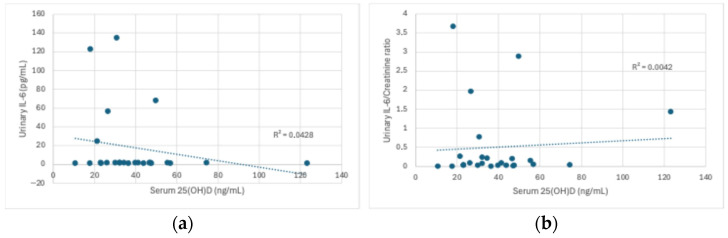
Linear regression plot for the correlation between: (**a**) vitamin D and urinary IL-6; (**b**) vitamin D and urinary IL-6/creatinine.

For the analysis of the influence of vitamin D on urinary concentrations of IL-6, LL-37, and NGAL, the Pearson correlation analysis does not support the existence of any association between vitamin D levels and urinary IL-6 (r = 0.2, r^2^ = 0.04, *p* = 0.32) or between vitamin D levels and the IL-6/creatinine ratio (r = 0.06, r^2^ = 0.004, *p* = 0.75). Figure 1a,b present the regression line plots for these correlations. The post hoc power analysis for the associations between serum vitamin D levels and the urinary IL-6/creatinine ratio revealed a very low statistical power of 4.8%.

**Figure 2:**
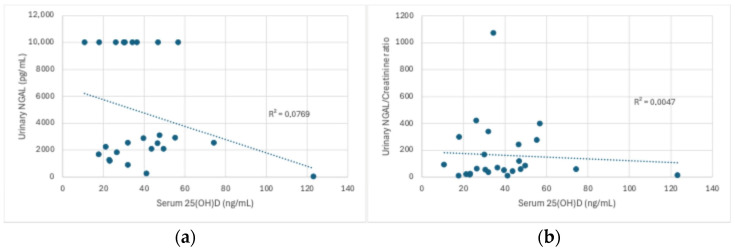
Linear regression plot for the correlation between: (**a**) vitamin D and urinary NGAL; (**b**) vitamin D and urinary NGAL/creatinine.

The analysis of the influence of vitamin D on urinary NGAL concentrations revealed a weak correlation between the two (r = 0.27, r^2^ = 0.07, *p* = 0.18). No correlation was detected between serum vitamin D levels and the urinary NGAL/creatinine ratio (r = 0.06, r^2^ = 0.004, *p* = 0.74). Figure 2a,b illustrate the regression line plots for these two correlations. The post hoc power analysis for the associations between serum vitamin D levels and the urinary NGAL/creatinine ratio revealed a very low statistical power of 5.03%.

**Figure 3:**
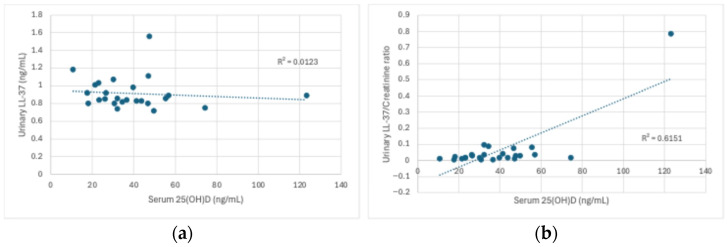
Linear regression plot for the correlation between: (**a**) vitamin D and urinary LL-37; (**b**) vitamin D and urinary LL-37/creatinine.

In evaluating the dependence of urinary LL-37 concentrations on serum vitamin D levels, the results did not reveal any correlation between the two (r = 0.11, r^2^ = 0.01, *p* = 0.59). The regression line plot for this analysis is shown in Figure 3a. The analysis of the correlation between vitamin D and urinary LL-37/creatinine revealed a strong correlation between the two (r = 0.78), with a moderate portion of the variation explained by the model (r^2^ = 0.61, *p* < 0.001). The linear regression plot for this correlation is presented in Figure 3b. The post hoc power analysis for the significant correlation between serum vitamin D levels and urinary LL-37/creatinine ratio indicated a statistical power exceeding 99.9%.

## 4. Discussion

Historically, vitamin D was empirically used to treat infectious diseases, particularly tuberculosis, through exposure to sunlight and supplementation with cod liver oil. Subsequent studies clarified its role in enhancing the immune response, including against Mycobacterium tuberculosis [4,17].

The present study builds upon the knowledge gained so far regarding the immunomodulatory role of vitamin D, with significant clinical implications for the frequency and severity of infectious processes, including those affecting the renal-urinary tract. Its impact on episodes of APNs, as well as its role in pro-inflammatory and anti-inflammatory immune mechanisms, suggests a possible involvement of vitamin D in the development of RS and the pathophysiology of RN. Investigating this potential involvement may provide useful information that could enable new approaches in the management of patients with VUR, with the ultimate goal of improving clinical outcomes for these patients.

At the level of the urinary system, Toll-like receptor (TLR) activation following contact with lipopolysaccharides in the bacterial cell structure represents the initial moment of triggering the innate immune response. After activation, TLR initiates a series of immune signaling mechanisms that lead to the development of the immune response. Among other effects, these signaling pathways stimulate the local production of 1-alpha-hydroxylase in macrophages and dendritic cells and enhance the expression of vitamin D receptors. This results in the local activation of vitamin D, which, through the overexpression of vitamin D receptors, induces the transcription of certain genes involved in the innate immune response. Thus, calcitriol stimulates the transcription of the gene encoding TLR2 and CD14, the latter being a cofactor of TLR4. Furthermore, vitamin D facilitates the interaction between antigen-presenting immune cells, which detect pathogens, and other cells of the immune system by stimulating the expression of certain cytokines, such as IL-8 or IL1β. Additionally, after binding to the vitamin D receptor, calcitriol induces the transcription of genes involved in the synthesis of AMPs (pathogen-associated molecular patterns), resulting in the production of innate immunity effectors such as LL-37 and defensins. 1,25-dihydroxyvitamin D can also induce autophagy, eliminating infected cells and facilitating bacterial clearance. To prevent the immune system from overactivation, calcitriol suppresses the production of pro-inflammatory cytokines, including IL-6. Therefore, the effectiveness of the innate immune response depends on the circulating levels of 25-hydroxyvitamin D [5,18,19].

In the literature, numerous studies have been published associating low serum levels of vitamin D with susceptibility to infections. A study conducted on 19,000 patients reported a higher frequency of upper respiratory tract infections in those with low vitamin D levels [20]. Similar results were reported following a study conducted on 800 military recruits in Finland [21]. There are also published studies reporting a greater susceptibility to developing lower respiratory tract infections in individuals with vitamin D deficiency [18]. Other studies have reported a connection between low vitamin D levels and infections with various pathogens, including the influenza virus, HIV, bacteria involved in vaginosis, and *Helicobacter pylori* [18].

Regarding the influence of low serum vitamin D levels on APNs, Tekin et al. conducted a study on 82 patients aged between 2 and 18 years who presented with UTIs without any risk factors. Their results showed that vitamin D deficiency is an independent risk factor for UTIs among pediatric patients. Comparing children with vitamin D levels below 20 nmol/L to those with sufficient vitamin D levels, they observed that those with a deficiency had a 3.5 times higher risk of developing UTIs [22]. Similar results, demonstrating that vitamin D deficiency is an independent risk factor for UTIs/APN in children, have been published by Shalaby et al., Sadeghzadeh et al., and Mahmoudzadeh et al. [23,24,25]. In their study, Yang et al. observed that patients with APN had lower vitamin D levels compared to those with lower UTIs [26]. Deng et al. conducted a meta-analysis that included nine individual studies, concluding that vitamin D deficiency is correlated with an increased risk of developing UTIs/APN [27]. It is worth noting that all these studies evaluated patients with a first episode of UTI/APN, without assessing the influence of vitamin D on recurrent UTIs/APNs. Nseir et al. published a study on the correlation between vitamin D and recurrent UTIs in a cohort of premenopausal women. The results showed that vitamin D deficiency is an independent risk factor for recurrent UTIs [28]. Muntean and Săsăran published a study reporting lower vitamin D levels in patients with recurrent UTIs compared to those who experienced only a single episode of UTI [29]. The results of our study regarding the correlation between serum vitamin D levels and APNs align with the findings of the previously mentioned studies. The lowest vitamin D levels were recorded in patients with more than five episodes of APN in their medical history. For these patients, the mean vitamin D level indicated a state of vitamin D insufficiency. In ascending order, the next values were observed in patients with 3–5 episodes of APNs, followed by those with two or fewer episodes. Thus, we observe that patients with lower vitamin D levels are more prone to a higher number of APN recurrences. Probably due to the small number of patients included in the study, the differences between the proposed groups did not reach statistical significance. For this reason, we consider it necessary to continue this analysis on larger patient cohorts. These findings highlight the potential role of vitamin D status as a modifiable factor in the prevention of recurrent APNs in children with VUR and support the rationale for further investigating vitamin D supplementation as part of targeted prophylactic strategies.

It was observed that the immune response, mediated through transcriptional mechanisms involving the vitamin D receptor, was significantly attenuated in individuals with 25-hydroxyvitamin D deficiency. This response was corrected through vitamin D supplementation [17]. Based on this observation, studies were conducted to investigate whether vitamin D supplementation significantly reduces the frequency of infectious episodes. One such study reported a 42% reduction in the incidence of influenza infections among school children who received vitamin D (1200 IU/day). The effect was more pronounced in those who had not previously received vitamin D [30]. Yamshchikov et al. published a review that included various clinical studies analyzing the use of vitamin D in the treatment or prevention of tuberculosis, influenza, and viral infections of the upper respiratory tract. Although the included studies showed considerable heterogeneity in terms of patient characteristics, cohorts, and methods, the results highlighted an important role of vitamin D in infectious pathologies, particularly in patients with vitamin D deficiency [31]. On the other hand, a study conducted on adult patients who were administered vitamin D (2000 IU/day for 12 weeks) did not identify any impact on the frequency or severity of upper respiratory tract infections [32].

Considering the results presented above, vitamin D supplementation for the prevention of recurrent UTIs has become a topic of interest in studies. Yang et al. reported, following a study conducted on young children, that vitamin D supplementation reduces the risk of UTIs [26]. Jorde et al. conducted a randomized controlled study with 511 prediabetic patients over five years to evaluate the role of vitamin D supplementation in UTI recurrences. The patients were divided into a group receiving vitamin D (20,000 IU/week) and a placebo group. During the study, 18 patients in the vitamin D-supplemented group experienced UTIs, compared to 34 patients in the placebo group, highlighting the role of vitamin D supplementation in reducing UTI recurrences [33]. In contrast to these results, the findings of Merrikhi et al. showed different outcomes. They conducted a similar study on pediatric patients with recurrent UTIs. Administration of 1000 IU of vitamin D daily for 6 months did not demonstrate a significant benefit in preventing UTI recurrences [34]. The differences in the reported results may be attributed to the varying doses of vitamin D supplementation, the durations of administration, the follow-up periods, and the baseline serum levels of vitamin D in the patients before the start of the study.

The protective effect of vitamin D regarding the development of UTIs and the prevention of recurrences is primarily due to its ability to stimulate the expression of urinary LL-37 [35]. Studies such as the one conducted by Nielsen et al. have demonstrated a significant association between urinary LL-37 levels and UTI incidence, with patients having low urinary LL-37 levels being more prone to developing UTIs [36].

Studies conducted on murine models have shown that in individuals unable to synthesize LL-37, the bacterial load and invasion in the urinary tract were much more severe. This aspect was not observed in subjects with induced neutropenia. However, once APN developed, neutropenic subjects exhibited more severe infections and higher mortality compared to subjects with LL-37 deficiency. These observations highlight the involvement of LL-37 in the early stages of the infectious process and immune response. Other reports of studies on murine models with LL-37 synthesis deficiency have included the development of severe systemic infections, sepsis, weight loss, and increased mortality [8,37].

In the context of the irrational use of antibiotic therapy and the selection of resistant bacterial strains, it is crucial to identify new therapeutic and prophylactic options. This is even more important for patients with VUR who require long-term antibiotic prophylaxis, a strategy that does not seem to impact the development of RS [38]. Due to their antibacterial properties and their sustained effectiveness over more than 1 million years of interaction with pathogens, various methods have been attempted for the therapeutic use of AMPs, including LL-37. The most commonly used methods have been oral and local administration at the site of the infectious process. It seems that oral administration of lactoferrin can reduce infection and inflammation in murine models infected with urinary E. coli [39]. The use of recombinant human LL-37 in the treatment of *E. coli* UTIs has shown antibacterial efficacy; however, its use as an antibacterial therapeutic agent requires caution. Concentrations far beyond physiological limits cause inflammation of the urinary tract epithelial cells and cell death. Intravesical administration of high concentrations of LL-37 resulted in edema and ulcerations at the mucosal level, with histopathological analysis revealing an inflammatory cellular infiltrate. Moreover, even at lower concentrations, cytotoxic effects and induction of apoptosis were observed [40,41].

Due to the aforementioned aspects, enhancing the immune response through the administration of immune modulators, such as vitamin D, could be a more effective alternative with fewer adverse reactions compared to the use of AMPs. Therefore, it is necessary to understand the correlation between vitamin D and urinary AMP expression, particularly LL-37. Our study revealed a strong correlation between serum vitamin D levels and urinary LL-37/creatinine values in patients with VUR. This could be the primary explanation for why patients with lower vitamin D levels in our cohort experienced more recurrences of APN.

The relatively small sample size represents an important limitation of the study, potentially reducing the statistical power to detect weaker associations. However, a post hoc power analysis conducted for the significant correlation between serum vitamin D and urinary LL-37/creatinine (r = 0.784, *n* = 25) revealed a power exceeding 99.9%, indicating that the study was adequately powered to detect strong associations. In contrast, for associations with weaker correlation coefficients—such as those between vitamin D and IL-6/creatinine (r = 0.065) or NGAL/creatinine (r = 0.069)—post hoc analysis showed very low statistical power (<5%). These non-significant findings should therefore be interpreted with caution, and larger studies are warranted to further explore these potential relationships. Additionally, no multivariable analysis was performed due to the limited sample size; therefore, the observed associations may have been influenced by unmeasured confounding factors.

The lack of correlation between uncorrected urinary LL-37 and vitamin D, contrasted with the strong correlation observed when using the LL-37/creatinine ratio, is most likely attributable to variability in urine dilution. As the urine samples were obtained from a pediatric population across a wide age range (4 months to 16 years), individual differences in hydration status, urinary output, and collection methods likely contributed to substantial variability in urine concentration. To account for this, we applied creatinine normalization to standardize biomarker levels—a widely accepted method in urinary biomarker research. This adjustment allows for more accurate comparisons across individuals and provides a more reliable reflection of true LL-37 excretion, thereby supporting the biological relevance of the observed correlation with vitamin D levels.

In their study, Övünç Hacıhamdioğlu et al. showed that pediatric patients with vitamin D deficiency cannot increase their urinary LL-37 levels during UTIs to the same extent as patients with sufficient vitamin D levels. This suggests that an adequate serum level of vitamin D is essential for optimal urinary LL-37 synthesis [42]. Hertting et al. observed that vitamin D supplementation significantly increased the production of LL-37 in bladder biopsy samples infected with E. coli, concluding that vitamin D supplementation could prevent UTIs [43]. Adams et al. demonstrated that a human subject’s ability to respond to an immune stimulus by increasing LL-37 concentration is directly proportional to the circulating level of 25-hydroxy vitamin D. Furthermore, this study showed that vitamin D supplementation (500,000 IU over five weeks) led to an increase in LL-37 synthesis [44].

The available evidence suggests that vitamin D supplementation may be particularly relevant in patients with VUR and vitamin D deficiency; however, prospective interventional studies are needed to determine whether supplementation can reduce APN recurrence and influence the development of renal damage.

In our study, patients with RS had a mean vitamin D level of 30.51 ng/mL, at the lower limit of the reference range for sufficient values. Although this value was lower than that of patients without RS, who had a mean level of 41.23 ng/mL, the difference between the two groups was not statistically significant. Previous studies on the involvement of vitamin D and its immunomodulatory mechanisms in developing RS have mainly focused on analyzing polymorphisms in the vitamin D receptor (VDR) gene. For instance, Aslan et al. demonstrated in their research that the ff genotype of the Fok1 polymorphism in the VDR gene is associated with a 3.95-fold higher risk of developing UTIs and RS compared to the FF phenotype among children with UTIs. Conversely, the Aa and aa genotypes of the Apa1 polymorphism appear to be protective factors [45]. Döven et al. evaluated in their study the correlation between serum vitamin D levels and the occurrence of RS secondary to APN. They concluded that vitamin D deficiency is a risk factor for developing RS in patients with recurrent APN, and vitamin D supplementation could provide clinical benefits to these patients [46]. Through its immunomodulatory role, vitamin D prevents an exaggerated pro-inflammatory response and exhibits antioxidant and anti-fibrotic activity [47]. These specific functions of vitamin D may exert a protective effect in preventing the development of RS and CKD by mitigating immune-mediated injury to renal tissues. While not conclusive, our findings align with prior evidence and suggest that vitamin D may play a role in modulating pathways involved in RS, warranting further physiopathologic and clinical research.

A major limitation of this study is the small number of patients analyzed, which makes it challenging to draw definitive conclusions regarding statistical significance. It limits the generalizability of the results and reduces the statistical power to detect moderate or weak associations. The cohort was derived from a specific subgroup meeting strict inclusion criteria, and as such, recruitment was inherently limited. Another important limitation is the cross-sectional observational design of this subgroup analysis, which precludes establishing causal relationships between vitamin D status, urinary biomarkers, and recurrent APN. An additional limitation could be the absence of a healthy control group. This restricts our ability to determine whether the observed immune marker levels are specific to children with VUR or reflect broader, population-level patterns. In addition, several factors known to influence vitamin D status, including seasonal variation, sunlight exposure, dietary intake, and vitamin D supplementation, were not systematically controlled for. Therefore, residual confounding may have influenced the observed associations between vitamin D levels, urinary biomarkers, and recurrent APN. Furthermore, several clinical factors known to affect the risk of recurrent urinary tract infections and renal injury in children with VUR, including VUR grade, bladder and bowel dysfunction, toilet training status, and circumcision status, were not systematically accounted for in the present analysis and may have contributed to residual confounding. In addition, potential influential outliers may have affected some observed associations, particularly given the relatively small sample size. Another potential limitation is the use of different urine collection methods according to patient age and bladder control. Although strict hygienic collection protocols were applied and patients with active urinary infection were excluded, variability in collection technique may still have influenced urinary biomarker measurements.

Nonetheless, the study provides preliminary insights into possible links between vitamin D and innate immunity in this high-risk group, which may inform the design of future controlled studies. Consequently, this study’s observations require confirmation through prospective, interventional studies on larger cohorts of patients with VUR.

## 5. Conclusions

By integrating the findings of the present study with previously published evidence, our results support a potential role for vitamin D in urinary tract innate immunity, possibly mediated through modulation of urinary LL-37 expression. Vitamin D may also contribute to the regulation of inflammatory responses involved in urinary tract infections and their renal complications. The observed association between serum vitamin D levels and urinary LL-37/creatinine supports further investigation of this pathway in children with VUR. Although no significant associations were identified between vitamin D levels and renal scars, reflux nephropathy, CKD stage, or APN recurrence, the observed trends and biological plausibility warrant further evaluation in larger prospective studies. Patients with vitamin D deficiency may represent a particularly relevant population for future interventional studies assessing the potential clinical benefits of vitamin D supplementation.

## Figures and Tables

**Table 1:** Reference intervals for the interpretation of vitamin D values.

25-Hidroxyvitamin D (ng/mL)	Interpretation
<10	Severe deficiency
10–20	Deficiency
21–29	Insufficient
>30	Sufficient
>100	Toxicity

**Table 2:** The correlation between serum vitamin D levels and the number of APNs.

APNs	Vitamin D (ng/mL)					
	No.	Mean	Std. Deviation	Std. Error	95% Confidence Interval for Mean	
					Lower Bound	Upper Bound
0	3	49.20	6.77	3.91	32.37	66.02
1–2	6	56.36	33.83	13.81	20.86	91.87
3–5	7	36.62	17.54	6.63	20.39	52.84
>5	9	27.30	12.81	4.27	17.45	37.15
Total	25	39.51	22.69	4.53	30.14	48.88

**Table 3:** The correlation between serum vitamin D levels and the presence of renal scars.

Renal Scars	Vitamin D (ng/mL)					
	No.	Mean	Std. Deviation	Std. Error	95% Confidence Interval for Mean	
					Lower Bound	Upper Bound
Absent	20	41.23	24.04	5.24	30.28	52.17
Present	5	30.51	11.72	5.86	11.84	49.17
Total	25	39.51	22.69	4.53	30.14	48.88

## Data Availability

Data is contained within the article.

## Abbreviations

The following abbreviations are used in this manuscript:

AMP	Antimicrobial peptide
APN	Acute pyelonephritis
CKD	Chronic kidney disease
eGFR	Estimated glomerular filtration rate
IL-6	Interleukin-6
LL-37	Cathelicidin
NGAL	Neutrophil gelatinase-associated lipocalin
RN	Reflux nephropathy
RS	Renal scar
TLR	Toll-like receptor
UTI	Urinary tract infection
VUR	Vesicoureteral reflux
